# Atomistic determinants of co-enzyme Q reduction at the Q_i_-site of the cytochrome *bc*_1_ complex

**DOI:** 10.1038/srep33607

**Published:** 2016-09-26

**Authors:** Pekka A. Postila, Karol Kaszuba, Patryk Kuleta, Ilpo Vattulainen, Marcin Sarewicz, Artur Osyczka, Tomasz Róg

**Affiliations:** 1 Structural Bioinformatics Laboratory, Biochemistry, Faculty of Science and Engineering, Åbo Akademi University, Tykistökatu 6A, FI-20520 Turku, Finland; 2 Department of Chemistry and Biochemistry, University of California San Diego, 92093-0340 San Diego, CA, USA; 3 Department of Physics, Tampere University of Technology, P.O. Box 692, FI-33101 Tampere, Finland; 4 The Institute of Science and Technology, 3400 Klosterneuburg, Austria; 5 Department of Molecular Biophysics, Faculty of Biochemistry, Biophysics and Biotechnology, Jagiellonian University, Gronostajowa 7, 30-387 Kraków, Poland; 6 Department of Physics, University of Helsinki, P.O. Box 64, FI-00014, Helsinki, Finland; 7 MEMPHYS – Center for Biomembrane Physics, University of Southern Denmark, Odense, Denmark

## Abstract

The cytochrome (cyt) *bc*_1_ complex is an integral component of the respiratory electron transfer chain sustaining the energy needs of organisms ranging from humans to bacteria. Due to its ubiquitous role in the energy metabolism, both the oxidation and reduction of the enzyme’s substrate co-enzyme Q has been studied vigorously. Here, this vast amount of data is reassessed after probing the substrate reduction steps at the Q_i_-site of the cyt *bc*_1_ complex of *Rhodobacter capsulatus* using atomistic molecular dynamics simulations. The simulations suggest that the Lys251 side chain could rotate into the Q_i_-site to facilitate binding of half-protonated semiquinone – a reaction intermediate that is potentially formed during substrate reduction. At this bent pose, the Lys251 forms a salt bridge with the Asp252, thus making direct proton transfer possible. In the neutral state, the lysine side chain stays close to the conserved binding location of cardiolipin (CL). This back-and-forth motion between the CL and Asp252 indicates that Lys251 functions as a proton shuttle controlled by pH-dependent negative feedback. The CL/K/D switching, which represents a refinement to the previously described CL/K pathway, fine-tunes the proton transfer process. Lastly, the simulation data was used to formulate a mechanism for reducing the substrate at the Q_i_-site.

To maintain diverse and complex cellular functions such as reproduction, growth or movement, all living organisms rely on constant supply of energy. The fundamentals of this life-sustaining energy metabolism or bioenergetics are known to a large extent, but the mechanistic details of relevant enzymatic reactions are still being actively studied and debated on. The membrane-embedded cytochrome (cyt) *bc*_1_ complex (or complex III; Fig. 1A) is a crucial component of the respiratory and photosynthetic electron transfer chains sustaining the energy requirements of both eukaryotes and bacteria.

The cyt *bc*_1_ complex operation or Q-cycle (named for the substrate co-enzyme Q or ubiquinone) begins when the fully protonated substrate quinol (QH_2_; Fig. 1B) binds into the Q_o_-site, where it is oxidized, *i*.*e*. it gives away two electrons (e^−^) and protons (H^+^). One e^−^ is transferred to the prosthetic 2-iron 2-sulfur cluster of the iron sulfur protein subunit, which then passes it on to the heme *c*_1_ group in the cyt *c*_*1*_ subunit (Fig. 1A). Meanwhile, the other e^−^ is routed towards the heme *b*_L_ cluster and the heme *b*_H_ group in the cyt *b* subunit (Fig. 1A). At the Q_i_-site of the cyt *bc*_1_ complex, the non-protonated substrate quinone (Q; Fig. 1B) acquires consecutively two electrons and, in total, two protons from the negative (N) side of the bioenergetic membrane^1^,^2^,^3^.

In our prior study, the binding modes of two substrate forms, QH_2_ and Q, were determined at the Q_o_-site of the cyt *bc*_1_ complex of purple photosynthetic bacterium *Rhodobacter capsulatus* using atomistic molecular dynamics (MD) simulations^4^. A highly coordinated water molecule was found to serve in a dual role both as a potential proton acceptor and as a structural gating mechanism for the short-circuit suppression. Similar arrangement was reported in a follow-up modelling study^5^. Likewise, coordinated water could also affect H^+^ transfers of the cyt *c* oxidase^6^,^7^.

The simulations also supported the X-ray crystallographic results by showing that cardiolipin (CL) has a conserved binding position close to the Q_i_-site (Fig. 1C)^8^,^9^. From this position the dianionic CL has been suggested to act as a H^+^ attracting antenna that feeds protons to Q being reduced at the Q_i_-site (Fig. 1C)^10^. The CL would donate protons first to Lys251 of the cyt *b* subunit, which then passes them to a string of interconnected water molecules leading up to the Q_i_-site (Fig. 1D). Utilizing the protons supplied by the CL/K pathway (Fig. 1C,D), a Q molecule is reduced to the fully protonated QH_2_ (Fig. 1B) *via* a reaction intermediate radical semiquinone (SQ; Fig. 1B). Again, lipids have been suggested to play a similar role in the H^+^ transfers of subunit III in the cyt *c* oxidase^11^.

There exist plenty of X-ray crystal structures showing the bound substrate at the Q_i_-site (Figs 1D and S1; Table S1; see Supplementary Information (SI)), but the exact reduction steps are unknown due to lack of structural data on explicit protons (Fig. 1B). To address this issue, the bacterial cyt *bc*_1_ complex was studied afresh using explicitly set up MD simulations (Table 1). Because only the anionic SQ has been detected using frozen electron paramagnetic resonance experiments at the Q_i_-site^12^,^13^,^14^,^15^,^16^, the substrate has been presumed to acquire both of the electrons (dianionic state) before accepting the two protons concomitantly^17^. While this scenario is possible, the other option is that the proton transfers are tightly coupled to separate e^−^ transfers (Fig. 1B) as recently suggested by quantum mechanics calculations^16^. Accordingly, the half-protonated SQ (Fig. 1B) could be formed prior to the second e^−^ transfer, but it has not been detected so far for example due to its inherent lability or short duration.

The results presented and discussed in this paper suggest that the binding of half-protonated/neutral SQ would be more stable and coordinated than that of Q and that there would be a clear advantage in reducing the C1-group for coordinating the C4-carbonyl H-bonding with His217. Importantly, the conserved residues Lys251 and Asp252 (Figs S1 and S2; Table S1) can form a salt bridge that meets the geometry criteria for a direct H^+^ transfer. Thus, instead of relying on an interconnected string of water molecules for the H^+^ transfers (Fig. 1C,D), the simulations indicate that the CL/K pathway ends in Lys251 rotating into the Q_i_-site and forming a salt bridge with Asp252. The empirical pKa calculations indicate that a direct H^+^ transfer could happen between the two ionizable residues and the lysine rotation-based H^+^ transfer would be pH-dependent. Moreover, the e^−^ transfer from the heme *b*_H_ group to Q slows down when Lys251 is substituted with residues unable to participate in H^+^ transfers^18^,^19^.

The explicitly set up simulations provide an unprecedented opportunity for reviewing prior site-directed mutagenic and X-ray crystallographic results (Table S1) regarding the Q_i_-site. Mechanistically the main finding of this work is the discovery of Lys251 rotation-based proton shuttle, which is a tangible refinement to the prior CL/K pathway hypothesis^10^. However, based on the simulations the inward rotation of Lys251 not only fine-tunes the H^+^ transfer process into the Q_i_-site, but potentially influences also the substrate binding/unbinding, reduction, and could even curb the aging-related superoxide generation. Accordingly, the accumulated data was used to formulate a novel Q reduction model in which the e^−^/H^+^ transfers are tightly coupled and sequential by nature as opposed to the previously suggested concomitant model^17^.

Figuring out the details of the reduction reaction at the Q_i_-site is a necessary step for achieving complete understanding of the cyt *bc*_1_ complex Q-cycle and, on a larger scale, the electron transfer chain itself.

## Results and Discussion

### The substrate binding requirements at the Q_i_-site

**Figure 1:**
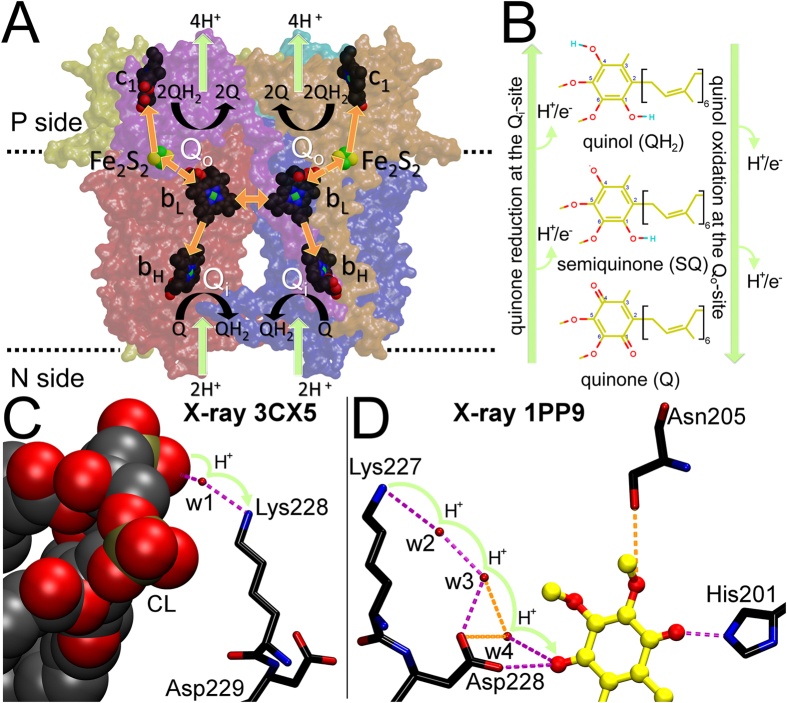
The cytochrome *bc*_1_ complex, proton/electron transfers of the Q-cycle, and the CL/K proton transfer pathway. (**A**) The dimer complex includes the cyt *b* (red/blue), cyt *c*_1_ (yellow/orange), and iron sulfur protein (ISP; cyan/magenta) subunits (PDB: 1ZRT)^22^. The Q_o_-site is located between the 2-iron 2-sulfur (Fe_2_S_2_) cluster and the low potential heme (*b*_L_). The Q_i_-site is adjacent to the high potential heme (*b*_H_). The arrows indicate the routes of the e^−^ (orange) and H^+^ transfers (green). (**B**) The arrows indicate the direction of the H^+^/e^−^ transfers during oxidation/reduction of the non-protonated Q, the half-protonated radical SQ and the fully protonated QH_2_ at the Q_o_- or Q_i_- sites, respectively. (**C**) In the CL/K pathway, lysine acquires a H^+^ from a cardiolipin (CL) molecule (PDB: 3CX5)^9^ and (**D**) passes it *via* a string of interconnected water molecules into the Q_i_-site to reduce the substrate (PDB: 1PP9)^20^. The H-bonds (≤3.4 Å) and possible bonds (≤3.6 Å) are shown with magenta and orange dotted lines, respectively. The CL-Lys251 interactions taking place at the membrane-protein periphery were considered in a previous MD simulation study^8^. The amino acid residues are shown as black sticks, substrate as yellow ball-and-stick representation and the heme bH group is shown as a CPK model.

**Figure 2:**
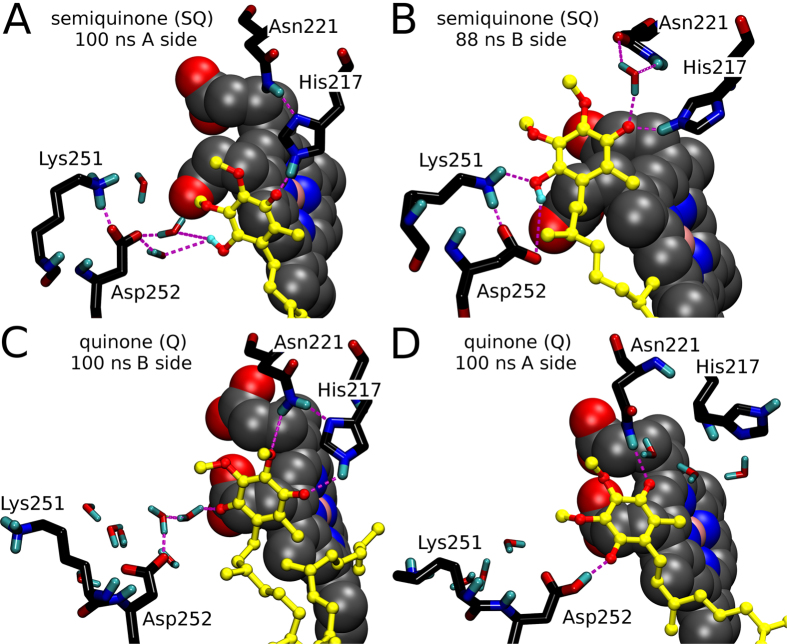
The binding modes of semiquinone and quinone at the Q_i_-site of cytochrome *bc*_1_ complex. (**A**) On the A side of the dimer, the C1-hydroxyl of neutral SQ (shown with yellow ball-and-stick representation) forms a water bridge with the Asp252^COO−^, while the C4-carbonyl H-bonds with the epsilon protonated His217. A Lys251^NH3+^-Asp252^COO−^ salt bridge is formed (conf_1_ in Table 1). The Asn221^NH2^ stabilizes the His217 positioning by H-bonding. (**B**) On the B side, the C4-hydroxyl of SQ H-bonds with both Lys251 and Asp252 that are forming a salt bridge (conf_1_ in Table 1). Both His217 and Asn221 H-bond with the C4-carbonyl of SQ. (**C**) On the B side, Q forms a water bridge with the Asp252^COO−^ and H-bonds with the His217 and Asn221 side chains that are also bonded to each other (conf_3_ in Table 1). The lysine assumes the outward rotamer pose. (**D**) On the B side, the C1-carbonyl of Q H-bonds with the Asp252^COOH^ and the As221^NH2^ H-bonds with the C4-carbonyl (conf_4_ in Table 1). Neutral Lys251 and Asp252 side chains are not bonding. For clarity only the polar protons are shown (cyan color). For further details see Fig. 1.

**Figure 3:**
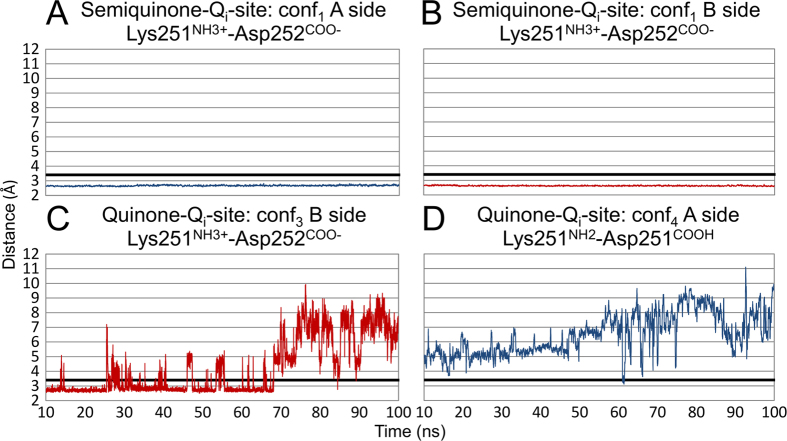
The Lys251-Asp252 salt bridge formation in the simulations. (**A**) The Lys251^NH3+^-Asp252^COO−^ salt bridge is formed on both A (blue line) and (**B**) B (red line) dimer sides in the conf_1_ (Table 1) simulation with neutral SQ bound at the Q_i_-site. (**C**) On the B side, Lys251 side chain assumes a clearly outward rotamer pose during the conf_3_ (Table 1) simulation with bound Q, although the side chain is initially forming a salt bridge with Asp252^COO−^ after the equilibration time. (**D**) On the A side, the neutral Lys251 and Asp252 side chains are not within bonding distance from the very beginning of the conf_4_ (Table 1) simulation with bound Q at the Q_i_-site. The H-bonding distance of 3.4 Å is indicated with a black line. For clarity, the results are shown as 10-point moving averages. The trajectory data shown in the A–D panels correspond to the substrate binding mode snapshots shown in Fig. 2A–D, where the salt bridge forming residues are shown.

The SQ/Q binding at the Q_i_-site is described thoroughly based on the MD simulations (Fig. 2) and X-ray crystallographic data in the SI (Fig. S1; Table S1); however, the binding requirements are summarized here:

(1) The C1-group of the substrate needs to form a H-bond or a water bridge with the Asp252 side chain (Figs 2; and S3A; Table S2). With bound neutral SQ at the Q_i_-site, the C1-hydroxyl would donate a hydrogen to the carboxylate group of Asp252 (or Asp252^COO−^; Fig. 2B). The opposite would happen with bound Q (or explicitly identical anionic SQ) when its C1-carbonyl would accept a hydrogen from the neutral Asp252 side chain (or Asp252^COOH^; Fig. 2D) or from a water molecule neighboring the Asp252^COO−^ (Fig. 2C).

(2) The His217 and/or Asn221 side chains H-bond with the C4-carbonyl of Q, in order to orient the substrate’s quinone ring to H-bond with the Asp252^COOH^. Unlike the X-ray crystallographic data (Fig. S1; Table S1), the simulations suggest that the Asn221 could H-bond with the C4-carbonyl at least momentarily in addition to interacting with the substrate’s C5-methoxy group (Figs 2C vs. D and S4).

(3) If the C1-group is reduced first (to produce neutral/half-protonated SQ), Lys251 side chain can rotate inward to form a lasting salt bridge with the Asp252^COO−^ (Figs 3A,B and S3; Table S2) and participate in the substrate binding (Fig. 2A,B and S3; Table S2). The Lys251^NH3+^ at the Q_i_-site helps to orient the quinone ring to assure H-bonding between the C4-carbonyl and the epsilon protonated His217 side chain. Accordingly, the neutral Lys251 points out of the Q_i_-site similarly as would happen with stably binding Q (Figs 2C,D and 3C,D).

(4) The C4-carbonyl of neutral SQ (and Q; see Step 2) needs to form a continuous H-bond with His217 (Figs 2A,B and S3; Tables S2–S3). This interaction, which would presumably be needed for the second set of sequential e^−^/H^+^ transfers (Fig. 1A,B), could be re-enforced by a H-bond between the amine group of Asn221 side chain and the delta nitrogen of His217 side chain (Fig. 2A,C).

### Lys251 functions as a switch-like proton shuttle between cardiolipin and Asp252

**Figure 4:**
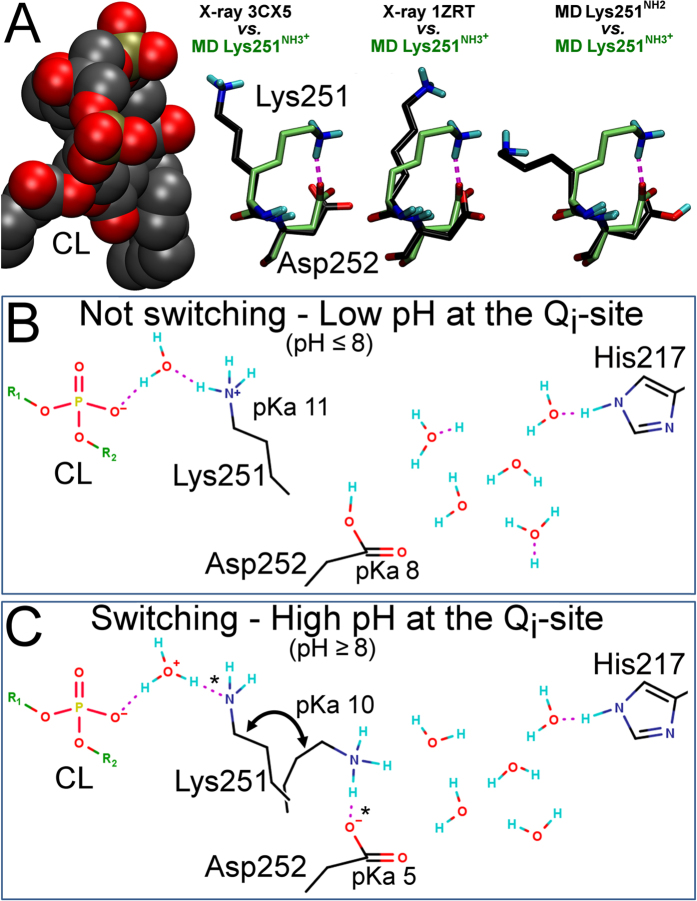
The switch-like operation of CL/K/D proton shuttle is pH-dependent. (**A**) The Lys251^NH3+^ forms a salt bridge with the Asp252^COO−^, when SQ is bound at the Q_i_-site. The rotation is extensive, if compared to the structure with peripheral cardiolipin (CL)^9^ or the initial pose^22^. If the inward pose of Lys251^NH3+^ is compared to the Lys251^NH2^ pose, it shows that the neutral lysine turns outwards. See Fig. 1 for further details. (**B**) The empirical pKa calculations indicate that the Asp252 side chain would be neutral, when the Lys251 side chain is out of the Q_i_-site (pKa value from 1SQP; Table S7). The Lys251^NH3+^ keeps the outward pose, when the Asp252 side chain is neutral, *i*.*e*. the switching does not happen, when the Q_i_-site is acidic. (**C**) The CL/K/D switching is triggered by the deprotonation of Asp252 side chain. The Lys251^NH3+^ rotates inwards and forms a salt bridge with the Asp252^COO−^. The empricial pKa calculations indicate that Lys251 and Asp252 are charged when forming a salt bridge (pKa value from 2FYN, G chain; Table S7). After a direct H^+^ proton transfer (*) from the Lys251^NH3+^ to the Asp252^COO−^, the neutral lysine rotates out to accept another H^+^ from the CL (*). The Asp252^COOH^ donates the newly acquired H^+^ to the solvent, if the pH rises at the Q_i_-site. This back-and-forth rotation of Lys251 would happen as long as the Q_i_-site was basic; ensuring also continuous protonation of His217.

The Lys251^NH3+^ can rotate directly into the Q_i_-site, form a salt bridge with the Asp252^COO−^ (Figs 3A,B and 4A and S3; Table S2), and participate in the half-protonated SQ binding either directly (Fig. 2B) or *via* a water bridge (Fig. 2A).

In the inward pose, the positive charge of Lys251 side chain matches the opposite properties of the Asp252^COO−^. The lysine forms a water bridge with the substrate in an X-ray crystal structure (1PPJ in Figs 1D and S1)^20^, but, notably, the salt bridge is seen in only one substrate-free mutant structure (PDB: 2FYN; chain G)^21^. The missing structure factors make it difficult to evaluate the PDB entry regarding the bridge. The inward pose of Lys251 is in marked contrast to the rotamer pose visible in the yeast cyt *bc*_1_ complex (PDB: 3CX5; Figs 1C and 4A)^9^, where the lysine is positioned close to the dianionic cardiolipin (CL; Fig. 4A). The close CL-lysine arrangement is found in altogether 17 X-ray crystal structures (Table S5). If Lys251 and Asp252 are set neutral, the lysine side chain turns more outwards in the simulation than in any prior structures (Figs 4A and S4B; Table S6).

When considering the Lys251 rotation (Figs 4A and S3; Tables S2–S3) in more depth, it seems that it is not only relevant for the substrate binding (Fig. 2A,B; Table S2) but that it could be of mechanistic importance as well (Fig. 4). In the substrate-bound structures and with the nonprotonated substrate Q in the simulations (Fig. 2C,D), the lysine resides out of the Q_i_-site (Fig. S1; Table S1) and it would therefore not be needed at least for the initial binding (Figs 2C,D and 3C,D; Table S1). Although the CL/K pathway could very well rely on water-mediated H^+^ transfers (Fig. 1C,D), we propose an alternative, simpler and more efficient mechanism. The lysine rotation could facilitate H^+^ transfers from the CL positioned in the periphery of the protein surface into the buried active site (Fig. 4B,C). The transfers into the Q_i_-site would rely solely on changing the protonation states of Lys251 and Asp252 and the lysine rotamer (Figs 4 vs. C; and 2A,B vs. C,D).

After the first e^−^/H^+^ transfers (Fig. 1A,B), there is no apparent reason for either Lys251 or Asp252 to be neutral, if neutral SQ is formed at the Q_i_-site. If Lys251 donates the first H^+^ to the Asp252^COO−^ in order to reduce Q, the neutral side chain should be able to rotate out and then return back in a fully protonated and positively charged state to reinforce the SQ binding (Figs 2A,B and 3A,B; Table S2). At any rate, the lysine side chain could reside at the site with the bound SQ long enough to ensure proper H-bonding between the C4-carbonyl and His217 (Fig. 2A,B) right after the first e^−^/H^+^ transfers (Fig. 1B). Then again, the salt bridge formation should not depend on the neutral SQ binding^20^. Thus, we propose that this salt bridge is formed and broken when a H^+^ is transferred into the substrate-free Q_i_-site or to promote stable SQ binding or even subsequent QH_2_ unbinding.

The back-and-forth repetition of the rotation would be consistent *in situ*, because the protonation of Lys251 and Asp252 would alternate between the two rotamers (Fig. 4B vs. C). A switch-like CL/K/D proton shuttle would function between the membrane periphery and the Q_i_-site without intermediary water molecules (Figs 1C,D vs. 4B,C). Although the Lys251 rotation probably happens at a subnanosecond time scale, even multiple successive water-mediated H^+^ transfers could be faster as a whole (0.93 ps/transfer)^22^. However, any potential timescale disadvantage of the switching (Fig. 4B–D) against the water transport model (Fig. 1C,D) would be overcome by the consistency and precise coordination of the Lys251 rotation inside the Q_i_-site (Fig. 4). The water-mediated H^+^ transfers are random or incoherent by nature, which reduces their overall efficiency.

### Negative feedback: pH-dependent switching shuttles protons to the Q_i_-site

Proton transfers require short distance between the ionizable groups and comparable pKa values for the residues^23^. The first requirement is met by the Lys251-Asp252 salt bridge formation (Figs 2A,B and 3A,B)^21^. The pKa values, derived using empirical calculations, indicate, quite reasonably, that Lys251 and Asp252 are charged, when forming the bridge (Table S7). The second requirement of comparable pKa values is observed in a majority of the X-ray crystal structures, furthermore, the residue pair has alternative titration states suggesting coupling (Tables S5 and S7)^24^. The outward pointing Lys251 side chain is generally charged; but the close presence of the peripheral CL increases this tendency.

The empirical pKa calculations predict that the Asp252 side chain is neutral with the bound substrate (Table S5), but the charged state would be possible without the substrate (Table S7). Both the charged and neutral Lys251 ultimately assume rotamer poses pointing out of the active site, when nonprotonated substrate Q binding was relatively coordinated (Figs 2C,D and S4; Tables S4 and S6). Based on these observations, it seems likely that in the substrate-bound X-ray crystal structures, where the lysine is pointing outward (Fig. S1; Table S1), the Q_i_-sites are occupied by non-protonated Q (or anionic SQ) together with the Asp252^COOH^ (Fig. S1; Table S1). Thus, the H^+^ transfer to the Q’s C1-carbonyl probably originates directly from the Asp252 (Fig. 2D) and without a bound substrate, the protons would be accepted by the solvent (Fig. 4B,C).

His217 is a logical proton source for the second reduction reaction with bound SQ (Fig. 1A,B) based on both the simulations (Fig. 2) and X-ray crystallographic data (Fig. S1; Table S1). The double protonation of His217 side chain (Table 1) would conveniently avoid the completely deprotonated state of imidazole ring during substrate reduction. However, this arrangement is not supported by the empirical pKa calculations (Tables S5 and S7) and, generally, it led to instability and/or the substrate unbinding in the simulations. The neutral SQ binding continued with the double protonated His217, only when the Asn221 side chain moved far away from its original position close to the bound substrate (conf_2_ at the B side in Fig. S3B; Table S3). This simulation result does not exclude the possibility that the double protonated His217 exist at least transiently *in situ*.

Overall, the empirical pKa calculations indicate that Lys251 could donate a H^+^ to the carboxylic acid of Asp252 side chain, which could then pass it along to the Q and *via* water to His217 (Fig. 4B,C). If the active site becomes basic enough, the Lys251^NH3+^ would rotate inward and donate a H^+^ to the Asp252^COO−^. This pH-dependent negative feedback is possible due to the unique position of the Q_i_-site at the protein-lipid interface (Fig. 1A).

### The CL/K/D switching hypothesis in the context of mutagenic studies

Site-directed mutagenesis experiments can produce precise data on the importance of specific residues for even the subtlest ligand-receptor interactions^25^,^26^,^27^,^28^. The conserved cyt *b* residues His217, Lys251 and Asp252 (Fig. S2) have been studied in prior mutagenic studies^18^,^19^, and this data is reassessed here in the light of the simulation results.

With H217A and D252A mutants for the cyt *bc*_1_ complex of *R. sphaeroides* (Fig. S2), the e^−^ transfer from the heme *b*_H_ to Q induced by a flash of light was blocked and, overall, the photosynthetic growth halted^18^. The loss of activity with H217A mutant results from the inability of alanine to form a direct H-bond with the substrate’s C4-carbonyl, although the CL/K/D switching should remain unaffected (Fig. S5A). Similarly, the D252A mutation should effectively prevent H-bonding between the residue and the substrate’s C1-group. The CL/K/D switching (Fig. 4B,C) should also be deactivated in the D252A mutant (Fig. S5B), because there is no polar attraction forcing the Lys251 side chain to rotate into the Q_i_-site (Fig. S5B).

The D252N, K251M and K251I mutations did not affect the photosynthetic growth but slowed down the e^−^ transfer from the heme *b*_H_ to Q^18^,^19^. With D252N mutant, this minor effect on the complex operation suggests that asparagine is able to H-bond with the substrate’s C1-group and also assist in water-mediated proton transfers to Q (Fig. S5C). The lysine could still rotate inward and even participate in the neutral SQ binding (Fig. S5C), albeit there is no strong electrostatic incentive for this. In contrast, the side chains of the mutated residues in the K251M and K251I mutants are hydrophobic and, thus, likely remain outside the Q_i_-site (Fig. S5D), where they cannot participate in the SQ binding or promote H^+^ transfers. In the absence of the Lys251 rotation-based proton shuttle (Fig. 4), the H^+^ transport from CL molecule to the Asp252 side chain would likely happen *via* interconnected water molecules (Fig. S5D).

On the one hand, the mutagenic studies clearly indicate that the CL/K/D switching would not be an on/off process but a subtler mechanism. When the proton shuttle is disabled (*e*.*g*. K251M mutant), water seems to be able to compensate for the lost function (Fig. 1C,D). On the other hand, the slowdown of the e^−^ transfer from the heme *b*_H_ to Q with the Lys251 mutants, the involvement of Lys251 in the neutral SQ binding in the simulations (Fig. 2A,B), and titration coupling (Tables S5 and S7) and overall conservation of the KD residue pair (Fig. S2) indicate that the mechanism would be an integral part of the Q_i_-site operation. The importance of protonable groups at positions 251 and 252 is emphasized by the observation that the photosynthetic growth is not blocked even, if the residues are swapped to produce K251D/D252K double mutant^19^. In fact, our recent studies employing combinations of single and double mutants suggest that a protonable residue is needed either at the 251 or 252 position to assure activity^29^.

Although the proposed mechanism is not an on/off process, it fulfills several hallmarks of a molecular switch. Firstly, the lysine side chain reversibly shifts between two stable states in and out of the Q_i_-site (Fig. 4A). Secondly, based on the empirical pKa calculations, the shifting happens in response to the pH level change (Fig. 4B,C). Thirdly, presence of a ligand (half-protonated SQ in Fig. 2A,B) at the site promotes the inward pose of Lys251 (Fig. 2A,B). Fourthly, although both the CL/K/D switching (Fig. 4B,C) and water (Fig. 1C,D) can shuttle protons to Q, only the lysine rotation and pairing/unpairing with the aspartate would be unequivocally directional and coordinated.

From evolutionary perspective, the inherent randomness of water-mediated H^+^ transfers is the logical reason why the lysine rotation-based proton shuttle involving dianionic CL would have evolved in the first place. The CL/K/D switching would increase the efficiency of the energy metabolism by fine-tuning the Q_i_-site operation of the cyt *bc*_1_ complexes (Fig. S2) and perhaps even boost the process in aberrantly low H^+^ concentration. It is likely that similar pH-dependent (K/D, D/K, K/D/K, K/D/K/D *etc*.) pathways involving protonable residues/lipids exist elsewhere as well.

### Proposed order of sequential quinone reduction at the Q_i_-site

**Figure 5:**
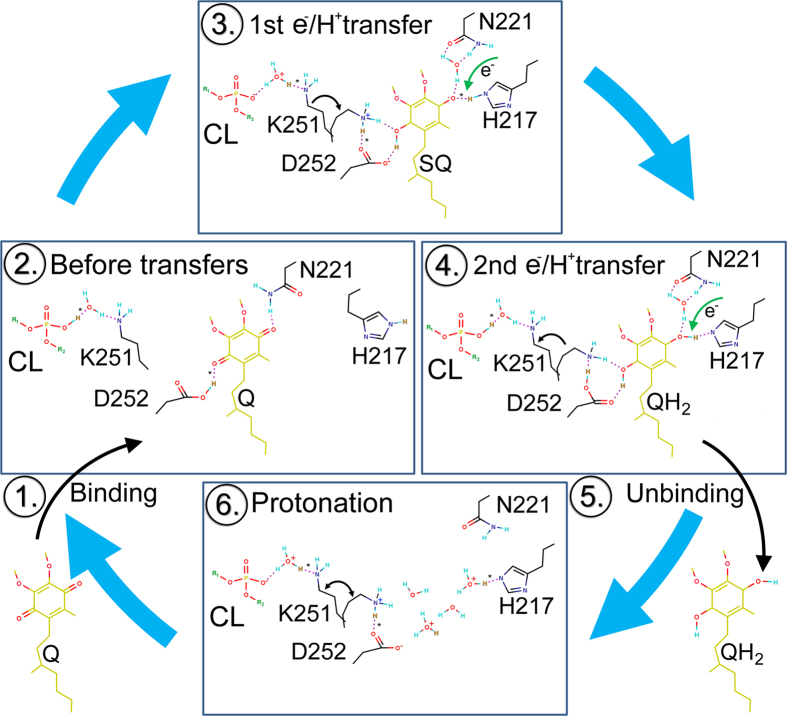
The proposed sequential quinone reduction mechanism at the Q_i_-site of the cyt *bc*_1_ complex. Those protons (H^+^) that are subject to transport (*) are shown in orange. For cardiolipin (CL) residing at the periphery is shown only the phosphate group. Note that the proton transfers between the peripheral CL and Lys251 do not necessarily involve water molecules. H-bonds are shown as magenta dotted lines. It is noteworthy that, if the two electrons are acquired separate from the H^+^ transfers (according to the concomittand proton transfer theory)^17^, it is unlikely that Lys251 rotation would play a role in the substrate binding (Fig. 5).

Based on the simulations (Fig. 2), prior mutagenesis experiments^18^ and X-ray crystallographic data (Tables S1,S5, and S7), the sequential Q reduction is suggested to happen accordingly (Fig. 5):

(1) Q binding is driven by the polar interactions between the quinone ring and the residues flanking the Q_i_-site.

(2) Upon Q binding, before e^−^/H^+^ transfers, the C1- and C4-carbonyl groups H-bond with Asp252 and Asn221 side chains, respectively. Note that the X-ray crystallographic data suggest that here His217 would eventually be H-bonding with the C4-carbonyl instead of the Asn221 side chain. At this point Lys251 would point out of the Q_i_-site (Fig. S1; Table S1).

(3) The first e^−^ transfer from the heme *b*_*H*_ to the substrate (producing anionic SQ) drives the H^+^ transfer to happen between the C1-carbonyl and the Asp252^COOH^. Alternatively, the H^+^ transfer could involve a water molecule residing between the aspartic acid and the C1-carbonyl (Fig. 2C). The Lys251^NH3+^ rotates into the Q_i_-site and forms a salt bridge with the Asp252^COO−^, which in turn stabilizes the positioning for the quinone ring of now neutral SQ by facilitating H-bonding and water bridge formation *via* the C1-hydroxyl and the C6-methoxy groups. At this point, the half-protonated SQ would be H-bonding stably with both the Asp252^COO−^ and the epsilon (or double) protonated His217, in other words, the quinone ring positioning would be fully coordinated.

(4) The H^+^ transfer could be reversible; *i*.*e*. the proton could move back-and-forth between Asp252^COO−^ and C1-carbonyl until the Lys251 side chain rotates inward and/or the second e^−^/H^+^ transfer ensues. In fact, only the deprotonated SQ has been detected at the Q_i_-site in previous experiments^12^,^13^,^14^,^15^. The stable binding of SQ could be required to wait for the second e^−^/H^+^ transfer and to prevent otherwise potentially rampant SQ-fuelled superoxide generation at the Q_i_-site. When the final e^−^ comes from the heme *b*_*H*_, the C4-carbonyl of half-protonated SQ takes a H^+^ from the His217 side chain. As a result, a fully protonated substrate QH_2_ is formed at the Q_i_-site.

(5) Finally, the reaction product QH_2_ unbinds. H-bonding between the Asp252^COO−^ and the C1-hydroxyl might postpone the H^+^ transfer from Lys251 to Asp252 for a while, but eventually the Lys251^NH3+^ could act as a “bouncer” that throws QH_2_ out of the Q_i_-site by donating a H^+^ to the Asp252^COO−^. The potential deprotonated state of His217 would also likely end quickly *via* water-mediated H^+^ transfers that also could promote the QH_2_ unbinding.

(6) At the outset, both His217 and Asp252 side chains are protonated and ready for Q binding. Lys251 would rotate in and out of the substrate-free Q_i_-site to upkeep this arrangement; transferring one H^+^ at a time from the peripheral CL molecule to the Asp252 side chain, which passes them to the solvent and via water also to His217 (Fig. 4B,C).

## Conclusions

The simulations indicate that the binding of half-protonated semiquinone (SQ) would acquire more coordinated binding pose than the deprotonated quinone (Q) at the Q_i_-site, because the Lys251 side chain participates in the neutral SQ binding (Fig. 2A,B). Thus, the substrate binding and H-bonding coordination could benefit from acquiring the protons sequentially shortly after each electron transfer. This sequential mechanism suggesting that the e^−^ transfers to be tightly coupled to H^+^ transfers should be tested in the future using for example quantum mechanics/molecular mechanics (QM/MM) calculations utilizing the new binding geometry seen in the simulations. The firmly coordinated binding of neutral SQ at the Q_i_-site, involving the inward rotamer of the Lys251 side chain, could be in part needed to curb the superoxide generation linked to aging-related cellular damage.

More importantly, in the fully bent rotamer pose (Fig. 4A), the positive lysine side chain forms a salt bridge with the negative Asp252 side chain (Figs 2A,B and 3A,B). The peripheral cardiolipin (CL) and the two ionizable residues are here suggested to function as a switch-like proton shuttle that transports protons from the membrane periphery directly into the Q_i_-site (Fig. 4B,C). Lys251 would acquire the protons from the CL molecule (Fig. 4A), but instead of relying on a string of interconnected water molecules (Fig. 1C,D)^10^, the lysine side chain rotation alone would shuttle the protons into the Q_i_-site (Fig. 4B,C). The Asp252 side chain would acquire the protons and pass them to the solvent. Upon Q binding the neutral Asp252 could H-bond with the C1-carbonyl and donate the H^+^ in response to the e^−^ transfer (see Step 3 in Fig. 5). The proposed CL/K/D switching and involvement of Lys251 in proton transfers in general is supported by the observation that the K251M mutation slows down the e^−^ transfer from the heme *b*_H_ group to Q^18^,^19^. The switching could assure constant protonation of Asp252 and *via* water also of His217 (Fig. 4B,C) that are likely primary proton sources during Q reduction (Fig. 5). In addition, the switching would be pH-dependent based on empirical pKa calculations, and thus the proton shuttle is activated only after the Q_i_-site becomes basic enough and protons are needed.

## Methods

The simulation protocol, force field derivation, and system build-up are presented in prior publications^4^,^8^,^30^,^31^. The dimer interface (PDB: 1ZRT)^32^ was filled with lipids that entered the cavity in a previous study (conf_4_ in ref. 8). The system compositions regarding the Q_o_- and Q_i_-site occupancies, redox centers states, and the substrate/residue protonation are shown in Table 1.

The molecular dynamics (MD) simulations were performed with NAMD2.9^33^ using the CHARMM 22 force-field and CMAP for the protein^34^, and the CHARMM 36 force field parameters for lipids^35^. The PME method was used for long-range electrostatics with a 12 Å cut-off^36^ for real vs. reciprocal space calculations. The time step was 1 fs. The target temperature and pressure were 310 K and 1 atm, respectively. Before running the 90 ns production simulations, specific angle and distance constraints were used during 10 ns equilibration simulations to keep the substrate’s C1- and C4-groups (Fig. 1B) within a H-bonding range with His217 and Asp252, respectively. The substrate binding was regarded as coordinated, if these two canonical interactions are formed (Fig. S1; Table S1).

The trajectory analysis and the 3D representations were prepared using BODIL^37^ and VMD1.9^38^. The 2D representations were made with MARVINSKETCH15.8.10.0 (2015, ChemAxon; http://www.chemaxon.com). The pKa predictions were done at pH 7.4 with default settings for the PDB structures using PROPKA3.1^39^,^40^ which uses an empirical approach to rapidly estimate the ionization state of protein groups. The predictive power of the software tool on the Q_i_-site residues was verified by analyzing selected snapshot structures with known protonation states (Table 1) extracted from the MD trajectories.

## Additional Information

**How to cite this article**: Postila, P. A. *et al*. Atomistic determinants of co-enzyme Q reduction at the Qi-site of the cytochrome *bc*_1_ complex. *Sci. Rep.* **6**, 33607; doi: 10.1038/srep33607 (2016).

## Supplementary Material

Supplementary Information

## Figures and Tables

**Table 1:** The simulation set-ups of the cytochrome *bc*_1_ complex.

MD simulation configuration^(1)^	Q_i_-site residue protonation^(2)^	Q_i_-site	Q_o_-site	heme *b*_L_^(3)^	heme *b*_H_^(3)^	heme *c*_1_^(3)^	Fe_2_S_2_ cluster^(3)^
conf_1_	Hse217, Lys251^NH3+^, Asp252^COO−^	SQ	Q	−2 red	−1 ox	−1 ox	−1 red
conf_2_	Hsp217, Lys251^NH3+^, Asp252^COO−^	SQ	Q	−2 red	−1 ox	−1 ox	−1 red
conf_3_	Hse217, Lys251^NH3+^, Asp252^COO−^	Q	QH_2_	−1 ox	−1 ox	−1 ox	0 ox
conf_4_	Hse217, Lys251^NH2^, Asp252^COOH^	Q	QH_2_	−1 ox	−1 ox	−1 ox	0 ox
conf_5_	Hsp217, Lys251^NH3+^, Asp252^COO−^	Q	QH_2_	−1 ox	−1 ox	−1 ox	0 ox
conf_6_	Hsp217, Lys251^NH2^, Asp252^COOH^	Q	QH_2_	−1 ox	−1 ox	−1 ox	0 ox

^(1)^Conf_1_-conf_2_ corresponds to conf_2_ and conf_3_-conf_6_ corresponds to conf_3_ in Postila *et al*.^4^, when considering the substrate binding and the redox center states. ^(2)^His217 side chain was either epsilon (Hse) or double (Hsp) protonated. Asp252 side chain was either neutral (Asp252^COOH^) or negatively charged (Asp252^COO−^). Lys251 was either neutral (Lys251^NH2^) or positively charged (Lys251^NH3+^). ^(3)^The formal charge of the redox centers: red = reduced and ox = oxidized.
