# Supplementary material for: Atomistic determinants of co-enzyme Q reduction at the Qi-site of the cytochrome bc1 complex
Source: Sci Rep. 2016 Sep 26;6:33607. doi: 10.1038/srep33607 (PMC5035994; doi:10.1038/srep33607)
Supplement: Supplementary Information [file srep33607-s1.pdf]

Supplementary Information for

Atomistic determinants of co-enzyme Q reduction at the Q_i-site of the cytochrome bc_1 complex

Pekka A. Postila[1, 2, 3], Karol Kaszuba[3,4], Patryk Kuleta[5], Ilpo Vattulainen[3,6,7], Marcin Sarewicz[5], Artur Osyczka[5], Tomasz Róg[3,6*]

[1] Structural Bioinformatics Laboratory, Biochemistry, Faculty of Science and Engineering, Åbo Akademi University, Tykistökatu 6A, FI-20520 Turku, Finland.
[2] Department of Chemistry and Biochemistry, University of California San Diego, 92093-0340 San Diego, CA, USA.
[3] Department of Physics, Tampere University of Technology, P.O. Box 692, FI-33101 Tampere, Finland.
[4] The Institute of Science and Technology 3400 Klosterneuburg, Austria.
[5] Department of Molecular Biophysics, Faculty of Biochemistry, Biophysics and Biotechnology, Jagiellonian University, Gronostajowa 7, 30-387 Kraków, Poland.
[6] Department of Physics, University of Helsinki, P.O. Box 64, FI-00014, Helsinki, Finland.
[7] MEMPHYS – Center for Biomembrane Physics, University of Southern Denmark, Odense, Denmark.

[*]Correspondence: tomasz.rog@tut.fi; +358 40 198 1179.

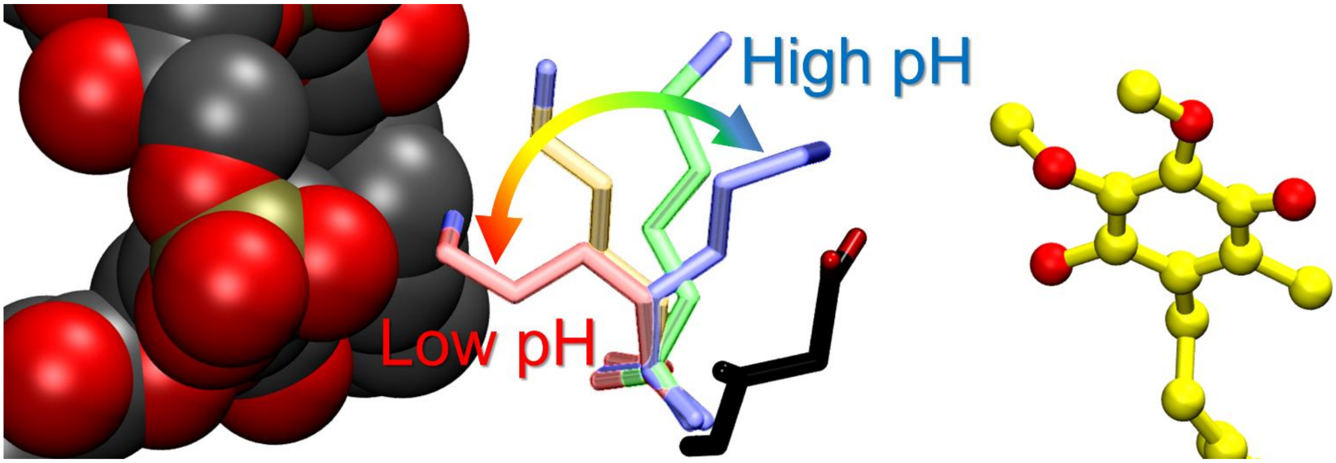

Table of Contents

Text S1. Semiquinone binds firmly to canonical residues at the Q_i-site

Text S2. Quinone binding does not promote Lys251 rotation inward

Table S1. Substrate binding distances at the Q_i-site in X-ray crystal structures of cytochrome bc_1 complexes

Table S2. Distances at the Q_i-site with semiquinone from the $conf_1$ simulation

Table S3. Distances at the Q_i-site with semiquinone from the $conf_2$ simulation

Table S4. Distances at the Q_i-site with quinone from the $conf_3$ simulation

Table S5. The empirical pKa values for the substrate-bound Q_i-site in the cyt bc_1 complex X-ray crystal structures

Table S6. Distances at the Q_i-site with quinone from the $conf_4$ simulation

Table S7. The empirical pKa values for the *apo* Q_i-site in the cyt bc_1 complex X-ray crystal structures

Figure S1. The substrate binding at the Q_i-site in the cyt bc_1 complex X-ray crystal structures

Figure S2. Cytochrome *b* sequence alignment at the Q_i-site for selected organisms

Figure S3. Distances between potential H-bonding partners at the Q_i-site with neutral semiquinone

Figure S4. Distances between potential H-bonding partners at the Q_i-site with nonprotonated quinone

Figure S5. The proposed effects of mutations on the CL/K/D switching and proton shuttling

Text S1. Semiquinone binds firmly to canonical residues at the Q_i-site

The reaction radical semiquinone (SQ) binding at the Q_i-site was robust in the simulation (Fig. 2A-B). This stability originated from the half-protonated state of the quinone ring and the charged state of Lys251 and Asp252 side chains ($conf_1$ in Table 1).

The C4-hydroxyl was able to H-bond with the $Asp252^{COO-}$, which in turn made it possible for the quinone ring to orient its C1-carbonyl to H-bond with the epsilon protonated His217 (Fig. 2B; Fig. S3A; Table S2). The connection between the C1-hydroxyl and Asp252 could also be water-mediated occasionally (Fig. 2A). Although Lys251 is not participating directly in the substrate binding in any of the X-ray crystal structures, the $Lys251^{NH3+}$ remained turned towards the Q_i-site with the bound SQ, where it H-bonded continuously with the $Asp252^{COO-}$ (Fig. S3; Table S2). On the B side of the dimer, Lys251 formed even a direct H-bond with the C1-hydroxyl (Fig. 2B; Fig. S3; Table S2).

The other major difference between the SQ simulation (Fig. 2A-B) and the previously reported X-ray crystal structures with the bound substrate (Table S1; Fig. S1) was the positioning of Asn221 side chain at the Q_i-site (Fig. 2A-B; Fig. S3; Table S2). The Asn221 is interacting favorably with the substrate's C6-methoxy group in the X-ray crystal structures (Fig. S1; also for Q in Fig. 2C). However, Asn221 also H-bonds with the C4-carbonyl either directly (Q in Fig. 2B) or it forms a water-mediated connection with SQ in the simulations (Fig. 2B). The Asn221 side chain is not necessarily competing with His217 for the substrate's C4-carbonyl (Fig. S3; Table S2), instead the H-bond donating residue (asparagine/serine/glutamine; Fig. S2) could be needed to coordinate His217 positioning.

Notably, the neutral SQ binding involves less waters on the B side than on the A side. Although the SQ could H-bond directly with the canonical residues, water can provide necessary flexibility for quinone ring positioning. Nevertheless, the relative stability of SQ binding at the Q_i-site is not unexpected. The reaction radical is known to reside at the Q_i-site from various experimental studies[1,2]. For comparison, it is still under contentious debate whether SQ is even momentarily formed during the oxidation reaction at the Q_o-site[1,3]. The sequential model (Fig. 5) dictates that the half-protonated substrate would stay in this stalemate state waiting for the second proton that can be acquired only after another electron has been dispatched into the Q_i-site (Fig. 1A-B). It would be very unfavorable, if the substrate could break its ties before acquiring both electrons/protons as the reaction radical SQ is linked to detrimental superoxide generation.

Text S2. Quinone binding does not promote Lys251 rotation inward

The quinone ring positioning or C1- and C4-group coordination for nonprotonated substrate Q at the Q_i-site (Fig. 2C-D) is less coordinated than that of neutral SQ.

When Lys251 and Asp252 side chains were set charged ($conf_1$ in Table 1), a salt bridge was formed during the equilibration phase of the simulations (0-10 ns) with Q at the Q_i-site. On the B side, the Lys251-Asp252 salt bridge broke during the restraint-free production simulation as water entered between the residues (Fig. S4A). On the A side, the salt bridge lasted throughout the simulation; however, this particular binding mode is not considered here, because it lacked any kind of C4-coordination (Fig. S4A; Table S4). On the B side of the dimer, the C1-carbonyl of Q H-bonded with His217. Meanwhile, the amine group of Asn221 side chain H-bonded with His217 and the C6-methoxy of Q. The C1-carbonyl formed a water bridge with the $Asp252^{COO-}$ (Fig. S4A). Thus, the logical conclusion is that without a H-bond donor at the C1-position, Lys251 would not be participating in the substrate binding.

The Q binding is less coordinated with the canonical residues (Fig. S2) than that of half-protonated SQ, even if the Lys251 and Asp252 side chains were set neutral ($conf_4$ in Table 1). The underlying thought behind this set-up was that the proton would be transferred between Lys251 and Asp252 prior to Q binding. On the B side, the $Lys251^{NH2}$ assumed quickly the outward rotamer pose and the C1- or C4-coordination was lost equally fast (Fig. S4B; Table S5). On the A side, the C1-carbonyl could H-bond with the $Asp252^{COOH}$, while the C4-carbonyl H-bonded with the Asn221 side chain (Fig. S4B; Table S5). Although Asn221 might be crucial for the initial stages of the Q binding, the overwhelming amount of X-ray crystallographic data suggest that the C4-carbonyl H-bonds to the His217 side chain instead (Fig. S1; Table S1).

The simulations contain variability regarding Q binding (Fig. S4). Only with bound SQ both Asp252 and His217 side chains could H-bond directly with the C1- and C4-groups simultaneously (Fig. 2B; Fig. S3; Table S2). The difference between the SQ and Q binding modes must arise mainly from the Lys251 protonation state and rotamer pose changes. The outward pose of the Lys251 side chain is preferred, when Q binding is at least partially coordinated at the Q_i-site (Fig. 2C-D; Table S4). Accordingly, the simulations imply that the inward pose of Lys251 would be needed to stabilize the H-bonding between neutral SQ's C4-carbonyl and His217 (Fig. 2A-B).

Table S1. Substrate binding distances at the Q_i-site in X-ray crystal structures of cytochrome bc_1 complexes.

PDB	Chain	Species	CL [(1)]	Lys251-Asp252 [(2)]	Asp252-C1 [(2)]	Lys251-C1 [(2)]	Lys251-C6 [(2)]	His217-C4 [(2)]	Asn221-C4 [(2)]	Asn221-C5 [(2)]
1BCC	G	*G. gallus*	no	5.3	3.0	8.6	8.1	2.4	6.0	3.6
2BCC	C	*G. gallus*	no	5.5	3.3	9.2	8.6	2.7	4.8	2.6
3CWB	D	*G. gallus*	yes	7.2	3.6	10.3	9.8	2.3	5.3	2.7
	P			7.2	3.3	10.3	9.5	2.5	4.9	2.4
3H1H	C	*G. gallus*	yes	7.1	3.0	10.3	10.1	2.4	5.5	2.7
	P			7.1	2.9	10.2	10.2	2.3	5.6	2.9
3H1J	C	*G. gallus*	yes	6.9	2.9	9.6	9.1	2.2	5.4	2.6
	P			6.9	2.6	9.8	8.7	2.5	5.2	2.7
3H1K	C	*G. gallus*	yes	7.8	3.6	10.3	10.5	2.4	5.3	2.5
	P			7.8	3.3	10.2	10.3	2.5	5.2	2.5
3L70	C	*G. gallus*	yes	7.0	3.1	10.5	10.4	2.4	5.5	2.8
	P			7.1	3.3	10.5	10.6	2.4	5.5	2.7
3L71	C	*G. gallus*	yes	6.8	3.0	10.3	10.2	2.5	5.5	2.8
	P			6.8	3.2	10.4	10.6	2.3	5.5	2.8
3L72	C	*G. gallus*	yes	7.3	3.1	10.5	10.4	2.4	5.7	2.9
	P			7.1	2.9	10.2	10.1	2.5	5.2	2.6
3L73	C	*G. gallus*	yes	7.1	2.9	10.2	9.9	2.5	5.3	2.7
	P			7.1	3.3	10.3	10.3	2.2	5.4	2.7
3L74	C	*G. gallus*	yes	7.4	3.0	10.0	9.4	2.4	5.3	2.6
	P			7.3	3.0	9.8	9.4	2.5	5.5	2.7
3L75	C	*G. gallus*	yes	7.0	3.1	9.8	9.2	2.4	5.2	2.5
	P			7.0	3.1	9.8	9.3	2.4	5.4	2.7
3TGU	C	*G. gallus*	yes	7.2	3.2	10.6	10.4	2.4	5.3	2.5
	P			7.2	2.8	10.6	10.1	2.5	5.3	2.7
4U3F	C	*G. gallus*	yes	7.1	2.8	10.7	9.8	2.8	5.2	2.9
	P			7.1	3.3	10.5	10.3	2.3	5.9	3.4
1NTZ	C	*B. taurus*	no	5.2	2.5	6.7	5.5	3.9	4.0	2.7
1PP9	C	*B. taurus*	yes	7.4	2.7	10.5	9.8	2.3	6.4	3.7
	P			7.3	3.0	10.9	9.8	2.2	6.1	3.6
1SQV	C	*B. taurus*	no	5.8	2.5	7.8	6.5	2.7	5.0	3.2
1SQQ	C	*B. taurus*	no	5.7	2.7	7.0	6.7	3.6	5.8	2.4
1SQX	C	*B. taurus*	no	5.4	2.7	7.8	7.2	2.8	6.0	3.4
2YBB	c	*B. taurus*	yes	7.3	3.0	10.9	9.8	2.2	6.1	3.6
	C			7.4	2.7	10.5	9.8	2.3	6.4	6.4
1KB9	C	*S. cerevisiae*	yes	7.9	3.6	10.4	9.8	5.6	5.8	3.3
1EZV	C	*S. cerevisiae*	no	8.0	3.7	9.9	9.7	5.7	6.1	3.2
1P84	C	*S. cerevisiae*	yes	8.0	3.1	9.9	9.2	5.8	6.5	2.9
2IBZ	C	*S. cerevisiae*	no	8.0	3.4	10.4	9.6	5.7	5.8	3.2
4PD4	C	*S. cerevisiae*	no	5.9	2.6	7.9	7.3	6.8	5.5	3.1
2QJY	A	*R. sphaeroides*	no	6.1	4.7	12.3	11.6	2.4	7.3	4.7
	D			8.1	4.7	14.5	13.0	2.2	6.6	4.5
	G			5.1	4.5	11.3	10.2	2.3	7.1	4.6
	J			9.4	5.5	15.7	14.2	2.4	6.7	4.9
	M			6.3	4.7	12.7	11.2	2.2	6.2	3.9
	P			6.1	3.8	11.4	10.4	2.3	6.7	4.4

(1) Cardiolipin (CL) head group (phosphate groups) in the general vicinity of the Lys251 side chain and the Q_i-site. (2) The residue numbering/naming from *R. capsulatus* (Fig. S2).

Table S2. Distances at the Q_i-site with semiquinone from the $conf_1$ simulation.

**H-bond partners(1)**	**AVG (Å)**	**MAX (Å)**	**MIN (Å)**	**MED (Å)**	**STDEV (Å)**	**H-BOND (%)(2)**
$Lys251^{NH3+}$-$Asp252^{COO-}$ A side	2.67	3.19	2.42	2.66	0.09	100.00
$Lys251^{NH3+}$-$Asp252^{COO-}$ B side	2.63	3.01	2.43	2.62	0.07	100.00
$Asp252^{COO-}$-$C1^{OH}$ A side	4.04	7.54	2.46	4.14	0.83	25.86
$Asp252^{COO-}$-$C1^{OH}$ B side	3.10	5.11	2.41	3.08	0.28	87.40
$His217^{NE}$-$C4^{CO}$ A side	2.87	6.46	2.51	2.82	0.22	97.83
$His217^{NE}$-$C4^{CO}$ B side	3.04	6.86	2.49	2.88	0.48	86.60
$Lys251^{NH3+}$-$C1^{OH}$ A side	6.93	10.34	4.27	7.18	1.07	0.00
$Lys251^{NH3+}$-$C1^{OH}$ B side	2.84	3.59	2.52	2.82	0.12	99.97
$Lys251^{NH3+}$-$C6^{OCH3}$ A side	4.91	8.18	2.68	5.12	0.97	9.31
$Lys251^{NH3+}$-$C6^{OCH3}$ B side	3.47	5.56	2.66	3.34	0.48	56.10
$Asn221^{NH2}$-$C4^{CO}$ A side	5.99	9.90	3.68	5.98	0.63	0.00
$Asn221^{NH2}$-$C4^{CO}$ B side	4.20	8.06	2.65	4.21	0.69	13.94
$Asn221^{NH2}$-$C5^{OCH3}$ A side	6.19	10.09	4.34	6.17	0.58	0.00
$Asn221^{NH2}$-$C5^{OCH3}$ B side	3.84	6.60	2.71	3.70	0.62	26.97
$Asn221^{NH2}$-$His217^{ND}$ A side	4.05	8.15	2.71	3.47	1.14	46.40
$Asn221^{NH2}$-$His217^{ND}$ B side	5.72	9.64	2.74	5.94	0.96	8.18

(1) The polar atoms of the H-bonding groups were used in the distance measurements. (2) Percentage of the simulation that a H-bond was formed. The upper limit for a H-bond was 3.4 Å. The H-bonding angle was not considered.

Table S3. Distances at the Q_i-site with semiquinone from the $conf_2$ simulation.

**H-bond partners(1)**	**AVG (Å)**	**MAX (Å)**	**MIN (Å)**	**MED (Å)**	**STDEV (Å)**	**H-BOND (%)(2)**
$Lys251^{NH3+}$-$Asp252^{COO-}$ A side	2.69	5.97	2.44	2.65	0.27	98.44
$Lys251^{NH3+}$-$Asp252^{COO-}$ B side	3.24	6.57	2.44	2.75	0.90	71.93
$Asp252^{COO-}$-$C1^{OH}$ A side	9.79	14.00	5.30	9.76	1.05	0.00
$Asp252^{COO-}$-$C1^{OH}$ B side	4.85	7.13	2.58	4.88	0.75	2.83
$Hsp217^{ND}$-$C4^{CO}$ A side	5.21	13.68	2.68	4.00	2.38	25.56
$Hsp217^{ND}$-$C4^{CO}$ B side	4.75	5.71	4.19	4.74	0.13	0.00
$Hsp217^{NE}$-$C4^{CO}$ A side	5.02	11.58	2.48	4.22	1.65	8.13
$Hsp217^{NE}$-$C4^{CO}$ B side	2.70	3.54	2.40	2.68	0.10	99.91
$Lys251^{NH3+}$-$C1^{OH}$ A side	12.97	17.39	9.18	12.77	1.30	0.00
$Lys251^{NH3+}$-$C1^{OH}$ B side	7.85	11.29	5.09	7.69	1.00	0.00
$Lys251^{NH3+}$-$C6^{OCH3}$ A side	10.55	15.42	6.61	10.20	1.57	0.00
$Lys251^{NH3+}$-$C6^{OCH3}$ B side	6.93	10.88	4.30	6.48	1.40	0.00
$Asn221^{NH2}$-$C4^{CO}$ A side	11.57	16.62	6.46	11.67	1.89	0.00
$Asn221^{NH2}$-$C4^{CO}$ B side	7.91	16.67	2.56	6.39	3.46	5.27
$Asn221^{NH2}$-$C5^{OCH3}$ A side	11.86	18.57	6.35	5.58	3.33	0.00
$Asn221^{NH2}$-$C5^{OCH3}$ B side	7.62	15.26	2.72	6.96	2.92	3.97

(1) The polar atoms of the H-bonding groups were used in the distance measurements. (2) Percentage of the simulation that a H-bond was formed. The upper limit for a H-bond was 3.4 Å. The H-bonding angle was not considered.

Table S4. Distances at the Q_i-site with quinone from the $conf_3$ simulation.

H-bond partners[(1)]	AVG (Å)	MAX (Å)	MIN (Å)	MED (Å)	STDEV (Å)	H-BOND (%)[(2)]
$Lys251^{NH3+}$-$Asp252^{COO-}$ A side	2.66	5.42	2.43	2.65	0.11	99.93
$Lys251^{NH3+}$-$Asp252^{COO-}$ B side	4.21	10.06	2.45	2.91	1.93	56.38
$Asp252^{COO-}$-$C1^{CO}$ A side[(3)]	4.79	7.40	2.79	4.76	0.74	1.71
$Asp252^{COO-}$-$C1^{CO}$ B side[(3)]	5.51	8.37	3.69	5.67	1.09	0.88
$His217^{NE}$-$C4^{CO}$ A side	5.44	11.38	2.61	5.30	1.21	5.02
$His217^{NE}$-$C4^{CO}$ B side	3.97	8.25	2.54	3.93	0.72	22.00
$Lys251^{NH3+}$-$C1^{CO}$ A side	7.31	10.55	4.16	7.27	1.00	0.00
$Lys251^{NH3+}$-$C1^{CO}$ B side	9.92	14.98	6.43	9.26	1.70	0.00
$Lys251^{NH3+}$-$C6^{OCH3}$ A side	5.80	9.61	3.29	5.69	0.95	0.01
$Lys251^{NH3+}$-$C6^{OCH3}$ B side	9.74	15.65	6.55	9.13	1.74	0.00
$Asn221^{NH2}$-$C4^{CO}$ A side	7.13	11.51	3.03	6.98	0.95	0.02
$Asn221^{NH2}$-$C4^{CO}$ B side	5.09	9.24	2.71	5.17	0.93	5.24
$Asn221^{NH2}$-$C5^{OCH3}$ A side	6.79	9.57	2.90	6.77	0.95	0.36
$Asn221^{NH2}$-$C5^{OCH3}$ B side	4.65	7.75	2.85	4.62	0.64	1.71
$Asn221^{NH2}$-$His217^{ND}$ A side	3.79	8.25	2.72	3.47	0.98	43.44
$Asn221^{NH2}$-$His217^{ND}$ B side	3.47	7.69	2.71	3.16	0.92	75.93

[(1)] The polar atoms of the H-bonding groups were used in the distance measurements. [(2)] Percentage of the simulation that a H-bond was formed. The upper limit for a H-bond was 3.4 Å. The H-bonding angle was not considered. [(3)] As both the carboxylate group of Asp252 and the C1-carbonyl of quinone are H-bond acceptors, a H-bond could not be formed between them, although the distance could occasionally meet the H-bonding criteria.

Table S5. The empirical pKa values for the substrate-bound Q_i-site in the cyt bc_1 complex X-ray crystal structures.

PDB	Chain	Species	CL [(1)]	Lys251 [(2)]	Asp252 [(2)]	His217 [(2)]	Lys251-Asp252 [(3)]
1BCC	C	*G. gallus*	no	8.35	6.14	5.07	2.21
2BBC	C	G. gallus	no	8.06 (6.61)	6.84 (8.29)	4.6	1.22
3CWB	D	*G. gallus*	yes	11.6	7.69	4.79	3.91
	P			11.04	7.54	4.25	3.5
3L70	C	*G. gallus*	yes	10.28	7.44	5.11	2.84
	P	*G. gallus*		10.24	7.47	5.23	2.77
3L71	C	*G. gallus*	yes	10.14	7.3	4.98	2.84
	P	*G. gallus*		10.19	7.52	5.13	2.67
3L72	C	*G. gallus*	yes	11.07	7.65	4.86	3.42
	P	*G. gallus*		11.04	7.53	5.08	3.51
3L73	C	*G. gallus*	yes	10.12	7.29	5.12	2.83
	P			10.24	7.33	5.16	2.91
3L74	C	*G. gallus*	yes	8.31	7.4	4.86	0.91
	P	*G. gallus*		9.03	7.32	4.44	1.71
3L75	C	*G. gallus*	yes	8.31	7.34	5.09	0.97
	P	*G. gallus*		8.18	7.28	4.53	0.9
3H1H	C	*G. gallus*	yes	10.15	7.3	4.7	2.85
	P			10.17	7.29	4.68	2.88
3H1J	C	*G. gallus*	yes	10.08	7.45	5.16	2.63
	P			9.99	7.47	4.91	2.52
3H1K	C	*G. gallus*	yes	10.51	8.44	4.52	2.07
	P			10.68	8.37	4.3	2.31
3TGU	C	*G. gallus*	yes	12.25	7.46	4.63	4.79
	P			12.24	7.37	4.99	4.87
4U3F	C	*G. gallus*	yes	11.97	7.44	4.61	4.53
	P	*G. gallus*	yes	11.76	7.69	4.25	4.07
1NTZ	C	*B. taurus*	no	8.17 (6.52)	6.96 (8.60)	4.97	1.21
1SQX	C	*B. taurus*	no	8.29	8.29	4.92	0
1PP9	C	*B. taurus*	yes	7.28	7.79	5.96	-0.51
	P			7.24	7.7	5.61	-0.46
1SQQ	C	*B. taurus*	no	8.09 (6.75)	7.33 (8.67)	5.22	0.76
1SQV	C	*B. taurus*	no	7.97 (6.78)	6.38 (7.56)	4.2	1.59
2YBB	c	*B. taurus*	yes	7.73	7.21	5.62	0.06
	C			7.27	7.25	5.96	0.02
1EZV	C	*S. cerevisiae*	no	6.96	7.52	4.87	-0.56
1KB9	C	*S. cerevisiae*	yes	7.47 (6.44)	7.65	4.83	-0.18
1P84	C	*S. cerevisiae*	yes	7.06 (6.05	7.31	4.71	-0.25
2IBZ	C	*S. cerevisiae*	no	6.95	7.35	4.87	-0.4
4PD4	C	*S. cerevisiae*	no	6.92 (5.74)	6.91 (8.09)	5.05	0.01
2QJY	A	*R. sphaeroides*	no	8.51 (7.50)	8.00 (9.02)	5.16	0.51
	D			8.67	8.5	5.31	0.17
	G			8.34 (6.63)	7.81 (9.52)	5.21	0.53
	J			8.87	8.83	5.33	0.04
	M			8.34	8.12	5.27	0.22
	P			8.04 (6.97)	7.99 (9.07)	4.85	0.05

(1) Cardiolipin (CL) head group (phosphate groups) in the general vicinity of the Lys251 side chain and the Q_i-site. (2) The residue numbering/naming from *R. capsulatus* (Fig. S2). The alternative titration state pKa values (PROKA3.1) are shown in parentheses. (3) The difference between Lys251 and Asp252 pKa values.

Table S6. Distances at the Q_i-site with quinone from the conf$_4$ simulation.

H-bond partners [1]	AVG (Å)	MAX (Å)	MIN (Å)	MED (Å)	STDEV (Å)	H-BOND (%) [2]
Lys251^{NH2}-Asp252^{COOH} A side	6.50	11.57	2.73	6.33	1.51	0.79
Lys251^{NH2}-Asp252^{COOH} B side	6.88	10.41	3.31	6.60	1.21	0.02
Asp252^{COOH}-C1^{CO} A side	2.85	4.68	2.44	2.80	0.22	97.13
Asp252^{COOH}-C1^{CO} B side	5.14	8.00	2.49	5.25	0.87	7.88
His217^{NE}-C4^{CO} A side	8.90	12.89	3.17	9.27	1.68	0.01
His217^{NE}-C4^{CO} B side	5.33	10.10	2.75	5.13	1.04	0.27
Lys251^{NH2}-C1^{CO} A side	9.08	14.38	5.21	8.69	1.62	0.00
Lys251^{NH2}-C1^{CO} B side	12.43	17.00	5.31	12.38	1.53	0.00
Lys251^{NH2}-C6^{OCH3} A side	7.74	12.80	3.82	7.27	1.64	0.00
Lys251^{NH2}-C6^{OCH3} B side	11.20	16.16	2.87	11.13	1.83	0.04
Asn221^{NH2}-C4^{CO} A side	3.33	7.62	2.58	3.18	0.55	68.91
Asn221^{NH2}-C4^{CO} B side	5.11	11.43	2.60	5.28	1.28	10.38
Asn221^{NH2}-C5^{OCH3} A side	3.75	7.52	2.64	3.58	0.65	36.38
Asn221^{NH2}-C5^{OCH3} B side	4.07	9.95	2.70	3.63	1.20	36.61
Asn221^{NH2}-His217ND A side	7.53	11.04	4.94	7.34	0.97	0.00
Asn221^{NH2}-His217ND B side	6.16	12.28	2.73	6.37	1.75	12.71

[1] The polar atoms of the H-bonding groups were used in the distance measurements. [2] Percentage of the simulation that a H-bond was formed. The upper limit for a H-bond was 3.4 Å. The H-bonding angle was not considered.

Table S7. The empirical pKa values for the *apo* Q_i-site in the cyt *bc*$_1$ complex X-ray crystal structures.

PDB	Chain	Species	CL[1]	Lys251[2]	Asp252[2]	His217[2]	Lys251-Asp252 [3]
1BE3	C	*B. taurus*	no	8.73 (5.38)	6.79	4.90	1.94 (-1.41)
1BGY	C	*B. taurus*	no	7.93	6.68	4.75	1.25
	O			8.08	6.34	4.71	1.74
1L0L	C	*B. taurus*	no	8.41	7.03	4.62	1.38
1L0N	C	*B. taurus*	no	8.23	7.76	4.71	0.47
1NTM	C	*B. taurus*	no	8.27	6.66	4.62	1.61
1SQB	C	*B. taurus*	no	8.16	7.37	4.67	0.79
1SQP	C	*B. taurus*	yes	10.57	7.91	4.47	2.66
2FYU	C	*B. taurus*	no	8.23 (6.78)	6.79 (8.23)	4.55	1.44 (-1.45)
2FYN	A	*R. sphaeroides*	no	8.78	8.10	4.66	0.68
	D			8.02 (6.94)	7.64 (8.73)	4.61	0.38 (-1.29)
	G			10.33	5.43	4.64	4.90
	J			8.41 (7.11)	7.52 (8.82)	4.65	0.89 (-1.71)
	M			8.34 (7.29)	7.81 (8.87)	4.56	0.53 (-1.58)
	P			7.99 (6.78)	7.81 (9.02)	4.57	0.18 (-2.24)
1ZRT[4]	C	*R. capsulatus*	no	7.85 (6.54)	8.69 (10.00)	5.33 (4.16)	-0.84 (-3.60)
	P			7.82 (6.50)	8.68 (10.00)	5.31	-0.86 (-3.50)
1KYO	C	*S. cerevisiae*	no	7.23	7.25	4.03	-0.02
	N			7.14	7.07	3.85	0.07

[1] Cardiolipin (CL) head group (phosphate groups) in the general vicinity of the Lys251 side chain and the Q_i-site. [2] The residue numbering/naming from *R. capsulatus* (Fig. S2). The alternative titration state pKa values (PROKA3.1) are shown in parentheses. [3] The difference between Lys251 and Asp252 pKa values. If there are alternative values, also these differences are shown in parentheses. [4] Missing side chain atoms added using PRIME in MAESTRO (Schrödinger Release 2016-1).

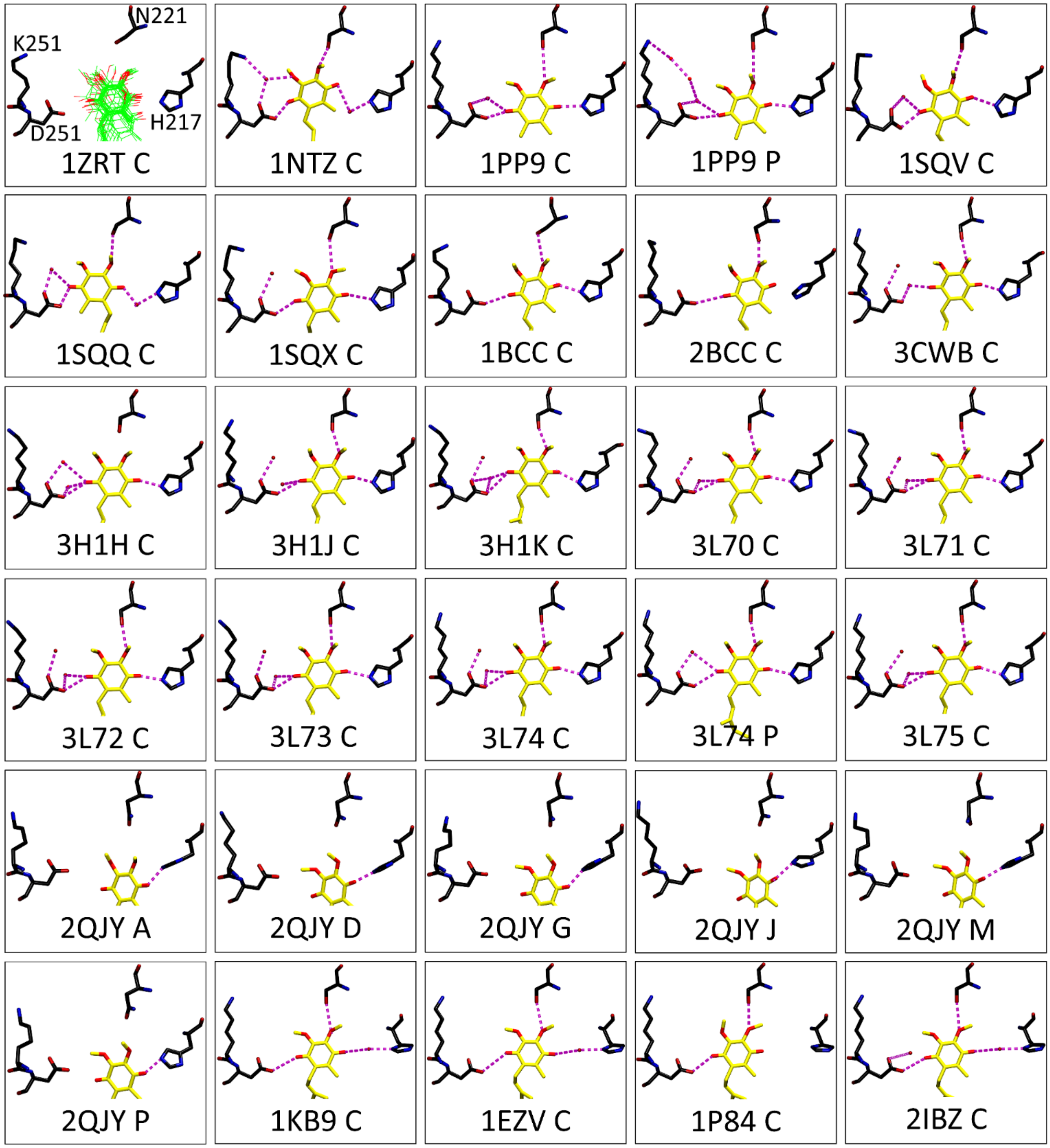

Figure S1. The substrate binding at the Q_i-site in the cyt *bc*$_1$ complex X-ray crystal structures. The residues (black sticks) and water molecules (red spheres), which interact with the substrate (yellow sticks) in the simulations are shown in the far up left corner for *R. capsulatus* (PDB: 1ZRT). The C^α atoms of the residues are superimposed and the substrate poses (green sticks) from the selected structures are shown at the Q_i-site of the original *apo* structure. Asparagine is substituted with serine for bovine and chicken structures (Fig. S2; Table S1). The limit for H-bonding is set to ~3.6 Å (magenta dotted line). Also the oxygen atom in the C5-methoxy group is shown to H-bond, although strictly speaking, it forms somewhat weaker electrostatically favorable interactions with asparagine or serine side chains instead.

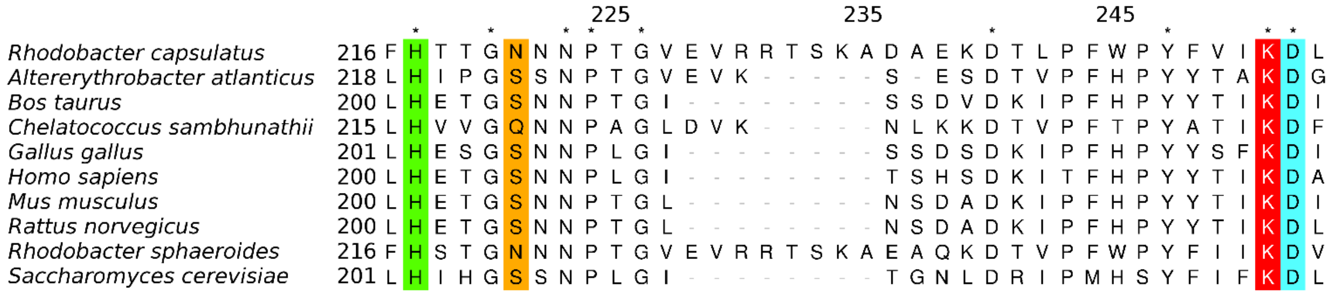

Figure S2. Cytochrome *b* sequence alignment at the Q_i-site for selected organisms. The cyt *b* subunit sequence alignment of bacterial and mammalian organisms shows that the Q_i-site residues His217 (green), Lys251 (red), and Asp252 (blue) are conserved (*; residues shown in Fig. S1). Although Asn221 is not conserved, the residue is substituted with either serine or glutamine that are both H-bond donors similarly as asparagine. The *R. capsulatus* (P0CY47), *A. atlanticus* (A0AF7KSN8), *B. Taurus* (P00157), *C. sambhunathii* (A0A0K6H0D3), *G. gallus* (P18946), *H. sapiens* (P00156), *M. musculus* (P00158), *R. Norvegicus* (P00159), *R. sphaeroides* (Q02761), *S. cervisiae* (P00163) sequences were acquired from the Uniprot (www.uniprot.org/).

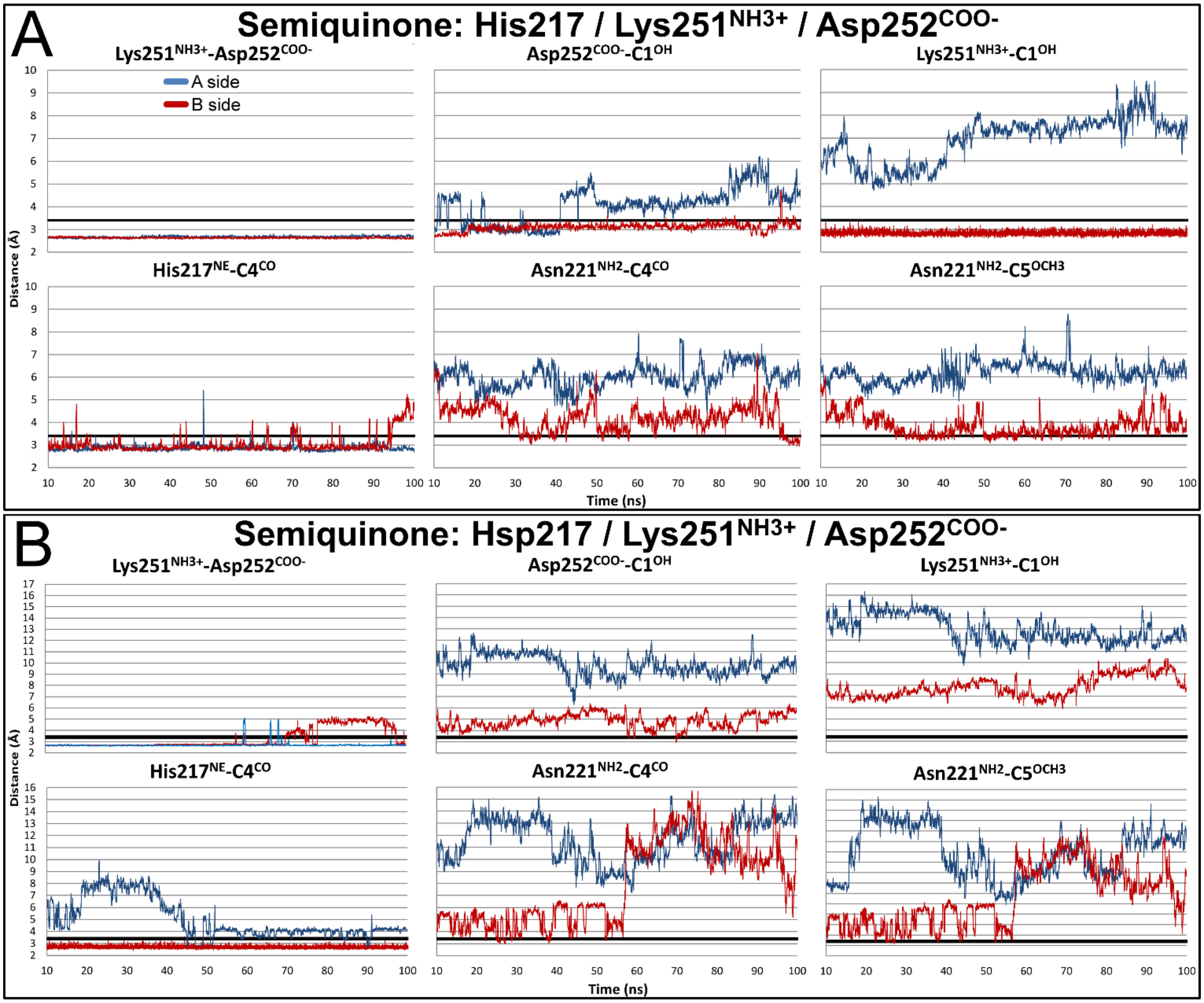

Figure S3. Distances between potential H-bonding partners at the Q_i-site with neutral semiquinone. A) The first set-up ($conf_1$ in Table 1) included the epsilon protonated His217 (or Hse217), positively charged Lys251 (or $Lys251^{NH3+}$), and negatively charged Asp252 (or $Asp252^{COO-}$). On the A side, the neutral SQ (blue lines) is forming more water mediated interactions than on the B side (red lines), which is reflected as longer distances for the interacting partners on the B side. Both A and B side arrangements assured good coordination for the C1- and C4-groups with the Asp252 and His217 side chains, respectively. Despite this, the H-bonding between His217 and the C4-carbonyl of SQ is clearly disturbed in the latter part of the simulation on the B side lacking water (red line). B) The second set-up ($conf_2$ in Table 1) included the double protonated His217 (Hsp217), the positively charged Lys251 (or $Lys251^{NH3+}$), and the negatively charged Asp252 (or $Asp252^{COO-}$). Note that on the A side (blue line) the C1-carbonyl is not H-bonding (or forming a water bridge) with the $Asp252^{COO-}$ (binding mode not discussed elsewhere), although both the C4- and C1-carbonyls are connected to His217 and Asp252 on the B side (red line), respectively. The H-bonding distance of 3.4 Å is indicated with a black line. For clarity, the results are shown as 10-point moving averages.

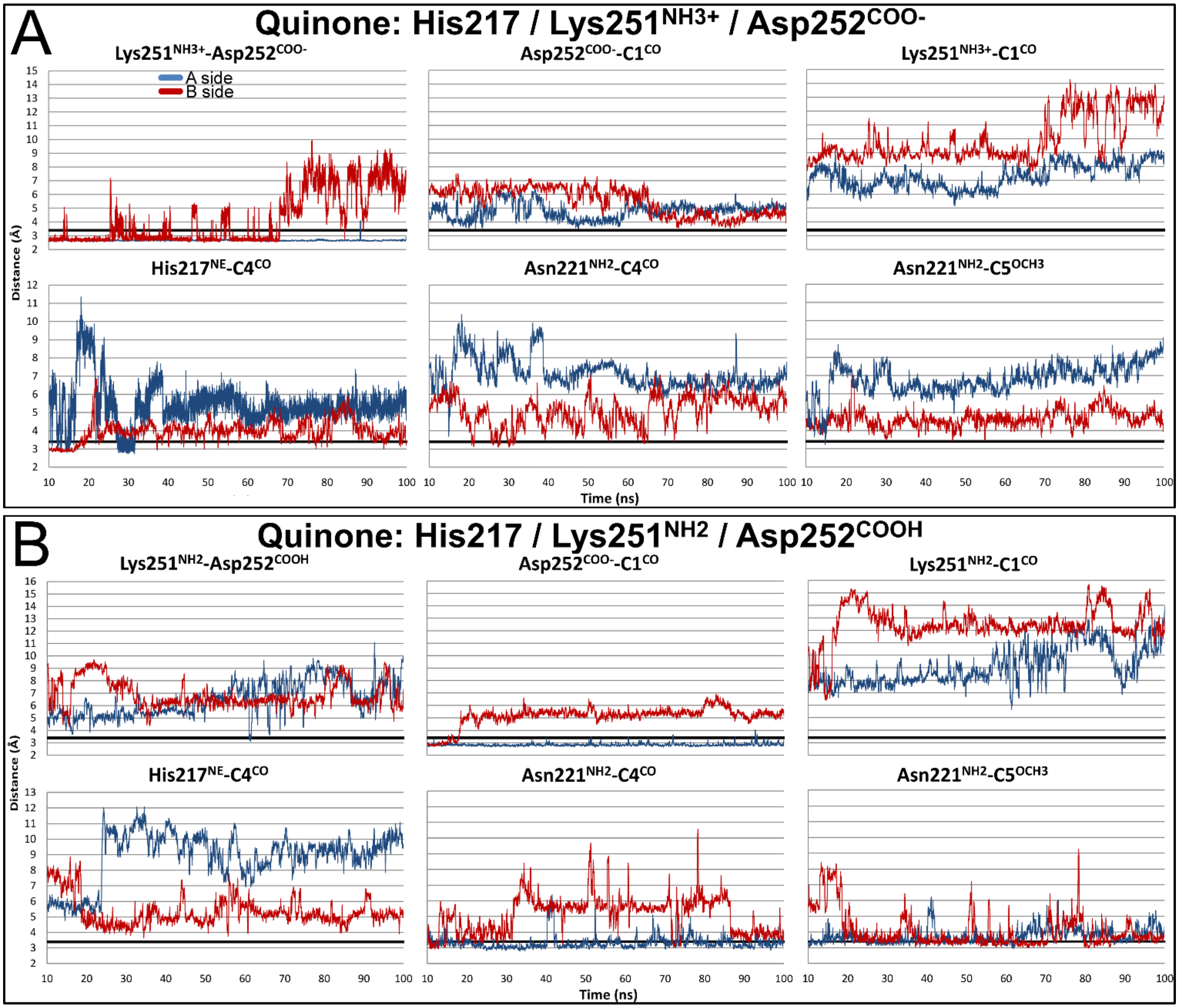

Figure S4. Distances between potential H-bonding partners at the Q_i-site with nonprotonated quinone. A) The first set-up included the epsilon protonated His217 (or Hse217), positively charged Lys251 (or $Lys251^{NH3+}$), and negatively charged Asp252 (or $Asp252^{COO-}$). On the A side, the C4-carbonyl of Q is not H-bonding (or forming water bridges) neither with His217 or the amine group of Asn221 side chain (or $Asn221^{NH2}$; blue lines; binding mode not shown elsewhere). In contrast, on the B side, His217 is H-bonding with the C4-carbonyl (red lines) as the $Asn221^{NH2}$ H-bonds with the histidine side chain and the C5-methoxy group (Fig. 2C). Accordingly, stable C1- and C4-group coordination resulted in breaking of the $Lys251^{NH3+}$-$Asp252^{COO-}$ salt bridge on the B side of the dimer with bound Q. B) The second set-up included the epsilon protonated His217 (or Hse217), the neutral Lys251 (or $Lys251^{NH2}$), and the neutral Asp252 (or $Asp252^{COOH}$). Only on the A side, the C1-carbonyl of was able to H-bond directly with the $Asp252^{COO-}$, and while in this position, the C4-carbonyl H-bonded with the Asn221 side chain instead of His217 (Fig. 2D). On the B side both the C1- and C4-groups were not connected to the Asn221, Asp252 or His217 side chains. The H-bonding distance of 3.4 Å is indicated with a black line. For clarity, the results are shown as 10-point moving averages.

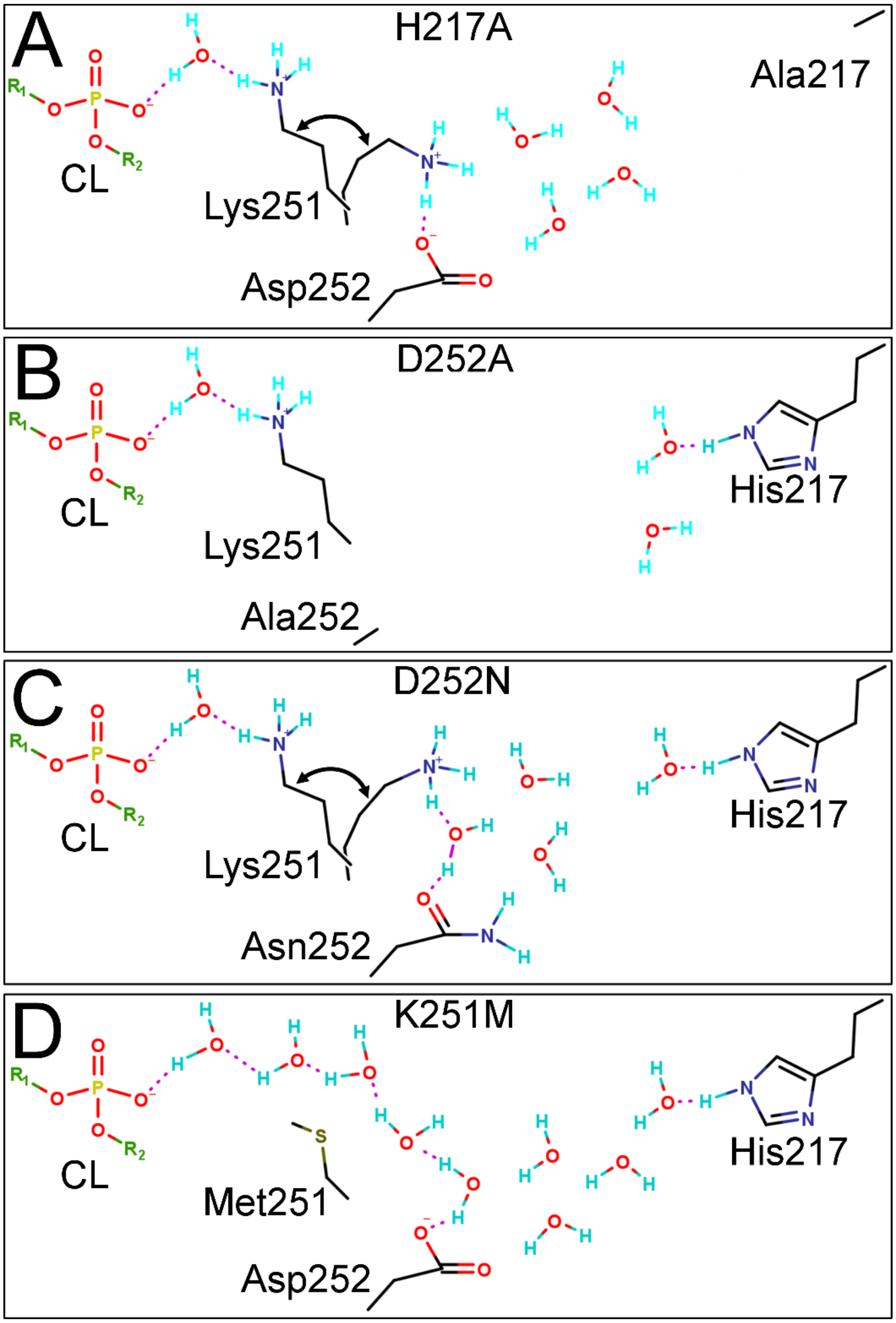

Figure S5. The proposed effects of mutations on the CL/K/D switching and proton shuttling. A) Mutating His217 to alanine (H217A mutant) is not expected to prevent the switching and proton shuttling, but alanine cannot H-bond or donate protons to the substrate (not shown). B) Substituting Asp252 with alanine (D252A mutant) is expected to make the Q_i-site more hydrophobic and prevent the switching of Lys251 between the cardiolipin (CL) and Asp252. Also binding should be weakened as the substrate is unable lock its C1-group at the site with the Ala252 side chain (not shown). C) With D252N mutant, the $Lys251^{NH3+}$ should be able to form water bridge or even a direct H-bond with the asparagine side chain (not shown). Although Lys251 could participate in the neutral SQ binding with D252N mutant, asparagine is unble to accept protons. D) Mutating Lys251 to methionine (K251M mutant) should deactive the K/D switching. The proton transfers would have to happen entirely *via* interconnected water molecules directly from the cardiolipin (CL).

References

1. Jünemann, S. Heathcote, P. & Rich, P. R. On the mechanism of quinol oxidation in the bc1 complex, *J. Biol. Chem.* **273,** 21603–21607 (1998).
2. Robertson, D. E. *et al.* Thermodynamic properties of the semiquinone and its binding site in the ubiquinol-cytochrome c (c2) oxidoreductase of respiratory and photosynthetic systems, *J. Biol. Chem.* **259,** 1758–1763 (1984).
3. Cape, J. L. Bowman, M. K. & Kramer, D. M. A semiquinone intermediate generated at the Qo site of the cytochrome bc1 complex: importance for the Q-cycle and superoxide production, *Proc. Natl. Acad. Sci. U.S.A.* **104,** 7887–7892 (2007).
